# Effect of Acidity Levels and Feed Rate on the Porosity of Aerogel Extracted from Rice Husk under Ambient Pressure

**DOI:** 10.3390/nano9020300

**Published:** 2019-02-20

**Authors:** Garram Ban, Sinae Song, Hong Woon Lee, Hee Taik Kim

**Affiliations:** 1 Department of Fusion Chemical Engineering, Hanyang University, 55 Hanyangdaehakro, Sangnok-gu, Ansan, Gyeonggi-do 15588, Korea; kdtry0@naver.com (G.B.); kkongbu@daega.co.kr (H.W.L.); 2 Department of Advanced Materials Science and Engineering, Hanyang University, 55 Hanyangdaehakro, Sangnok-gu, Ansan, Gyeonggi-do 15588, Korea; mokakid@hanyang.ac.kr; 3 Daega Powder Systems Co., LTD., Head office & Seoul factory, 22-31, Buil-ro 1na-gil, Guro-gu, Seoul-si 08262, Korea

**Keywords:** silica aerogel, rice husk, ambient pressure synthesis, rice husk aerogel

## Abstract

Silica aerogels have attracted tremendous interest due to their high specific surface area and the physical, chemical, and mechanical properties as promising materials for thermal insulation, chemical sensors, and energy storage devices. However, large-scale production of silica aerogels remains a challenge due to costly alkoxide precursors and energy-intensive supercritical drying processes. This paper analyzes the effect of acidity levels and feed rate on the porosity of rice husk aerogels with high specific surface area under ambient pressure. This synthetic approach is cost-effective, eco-friendly, and facilitates recycling. Rice husk ash, which consists of 92% amorphous pure silica, was produced by combustion. A process of solvent exchange and surface modification under ambient pressure at different pH levels was conducted for synthesis of the aerogel. The specific surface area of rice husk aerogel was confirmed as ranging from 385 to 861 m^2^/g under pH 1 to pH 9 and acid feed rate of 0.5 to 5.0 mL/min. The optimized aerogel had a specific surface area of 861 m^2^/g, a pore volume of 3.33 cm^3^/g, and an average pore diameter of 12 nm when synthesized at pH 1 and an acid feed rate of 2.5 mL/min. The aerogel was found to be highly hydrophobic, with a water contact angle of 156° up to about 340 °C.

## 1. Introduction

Silica aerogels are highly porous materials with a three-dimensional network of approximately 90% mesopores filled with air. The aerogels have been widely studied for potential applications in various fields due to properties such as high porosity (85%–99%), low density (0.03–0.3 g/cm^3^), high specific surface area (600–1000 m^2^/g), high transparency, low thermal conductivity (~0.015 W/m·K), low sound velocity (~100 m/s), low dielectric constant (~1.1), inflammability, and chemical stability [1]. They have particularly been investigated as thermal insulation materials to apply in the fields of building, industry, aerospace, and biomaterials. Aerogels have the properties of biomaterials, including large porosity, lightweight, a large internal surface, an interconnected three-dimensional structure, and biocompatibility [2]. The diverse properties of aerogels have prompted their applications in biotechnology, such as in tissue engineering, biomedical implantable devices, antibacterial materials, drug delivery, disease diagnosis, ultrasound contrast agents, and biosensing [3,4,5,6]. Despite their usage in many applications due to these attractive properties, large-scale production is limited due to the need for costly alkoxide precursors and energy-intensive supercritical drying processes.

The challenge of conventional aerogel synthesis in this study was overcome by extracting pure silica from rice husk (RH) using solvent exchange and drying it under ambient pressure. This alternative synthetic method provides economic benefits for the mass production of aerogels as the process is at low pressure, uses low-cost raw materials, and the solvent in the solvent exchange can be recycled. Surface modification is a prerequisite step in solvent exchange to remove the hydroxy group on the surface in order to maintain the formation structure of a silica gel [1]. The ambient pressure drying process is an alternative economical method in the synthesis of aerogels. Elimination of capillary stress during the drying process is the key factor in preparing aerogels. Capillary tension in a silica wet gel is generated from liquid evaporation under drying by the liquid–vapor interface and induces shrinkage of the gel and balance with the compressive stress in a solid network [7]. As a source of aerogel, untreated RH has many components, including silica, silicon carbide, silicon nitride, silicate, and zeolite, because of its high silica content. The outermost layer of paddy rice is composed of about 20 wt% paddy grain, and silica comprises 20 wt% of the husk [8]. The high content of silica is based on the superior ability of grain roots to uptake Si from soil [9,10,11]. Rice husk ash (RHA), which is produced as a by-product of combustion, contains about 90% amorphous silica.

The novelty of this research lies on describing the influence of acidity level on the porosity of aerogels extracted from only rice husk under ambient pressure and comparing this process with conventional aerogel synthesis [7,12,13]. The aim of this research is to develop an eco-friendly source of silica aerogels under ambient pressure using a cost-effective process with re-usable solvents.

## 2. Materials and Methods

### 2.1. Synthesis Process

Raw RH was obtained as a by-product after rice threshing from Geoje, Republic of Korea. Rice husk was calcined at 600 °C for 4 h to decompose the organic matter in the husk and turn it into RHA, which was used as the starting material for the extraction of silica. The sodium silicate solution was mixed with 6 g RHA in 90 mL 1 M sodium hydroxide beads 97% (Daejung Chemical Co., Siheung-si, Korea) and then heated at 90 °C for 2 h. After vacuum filtration of the mixture, transparent sodium silicate sol was obtained. The sodium silicate sol was titrated at an acidity level (pH 1 to pH 9) with 1.5 M hydrochloric acid at 35%–37% (Samchun Chemical Co., Seoul-si, Korea) at a constant feed rate (0.5 to 5.0 mL/min) using a peristaltic pump. The prepared silica gel was aged for 24 h at room temperature (RT). The aged wet gel was immersed in deionized water for 12 h at RT to remove the remaining salts in the gel structure. The hydrogel was subsequently immersed in an intermediate solvent (ethyl alcohol anhydrous 99.9% from Daejung Chemical Co., Siheung-si Korea) for 12 h at RT. The solvent exchange and surface modification process was conducted using n-hexane 95% (Daejung Chemical Co., Siheung-si Korea) as a nonpolar solvent and trimethylchlorosilane and chlorotrimethylsilane (TMCS) ≥98% (Sigma-Aldrich/Merck KGaA, Darmstadt, Germany) as the silylating agent. The centrifuged silica gel was immersed in 10% *v*/*v* TMCS in n-hexane for 24 h at RT, and the solution was removed by vacuum filtration. The surface-modified silica gel was dried at 60, 80, 120, and 150 °C for 3, 3, 2, and 2 h step-by-step.

### 2.2. Characterization Methods

Thermogravimetric (TG), derivative thermogravimetric (DTG), and differential thermal analysis (DTA) were performed to confirm the thermal decomposition of rice husk and to determine the ideal calcination temperature of rice husk using TG-DTA2000SA (Bruker, Germany). About 6 mg of RH was heated from 30 to 1000 °C in air with a heating rate of 10 °C/min. The chemical composition of RHA was investigated by X-ray fluorescence (XRF) using ZSX Primus II (Rigaku, Tokyo, Japan). The amorphous nature of silica in RHA was verified using X-ray diffraction (XRD; D/MAX-2500/PC, Rigaku, Tokyo, Japan). Concentrations of elements in sodium silicate from RHA were measured using inductively coupled plasma atomic emission spectroscopy (ICP-AES; Optima 4300DV, PerkinElmer, Waltham, MA, USA). The textural properties of rice-husk-based silica aerogel were analyzed by physical adsorption of nitrogen at 77 K using Tristar II (Micromeritics, GA USA). The specific surface area of the aerogel was determined by the Brunauer–Emmett–Teller (BET) method. Pore volume and average pore size were evaluated by the Barrett–Joyner–Halenda (BJH) method based on the desorption isotherm. All samples were degassed at 250 °C for 3 h before nitrogen adsorption. The porous structure of the silica aerogel was observed using field emission scanning electron microscopy (FE-SEM; S-4800, Tokyo, Japan) and transmission electron microscopy (TEM; JEM-2100F, Jeol, Tokyo, Japan). The size of the silica particles was measured from the FE-SEM image using Imagetool. The functional groups of the surface-modified silica aerogel were analyzed by Fourier transform infrared spectroscopy (FTIR) in a wavenumber range of 400–4000 cm^−1^ using a Nicolet iS5 FTIR spectrometer (Thermo Scientific, MA USA). For the FTIR analysis, the aerogel powder was well mixed with potassium bromide and pressed into a pellet. The contact angle was measured to determine the degree of hydrophobicity of the silica aerogel using the SmartDrop Lab (Femtofab, Seongnam-si Korea). Water droplet of about 9 μL was placed on the surface of the aerogel, and the contact angle was measured. The thermal stability of the silica aerogel was confirmed by TG and DTA using TG-DTA2000SA (Bruker, Billerica, MA, USA). A 5 mg quantity of aerogel powder was heated from 30 to 1000 °C at a heating rate of 5 °C/min in air.

## 3. Results and Discussion

**Figure 1:**
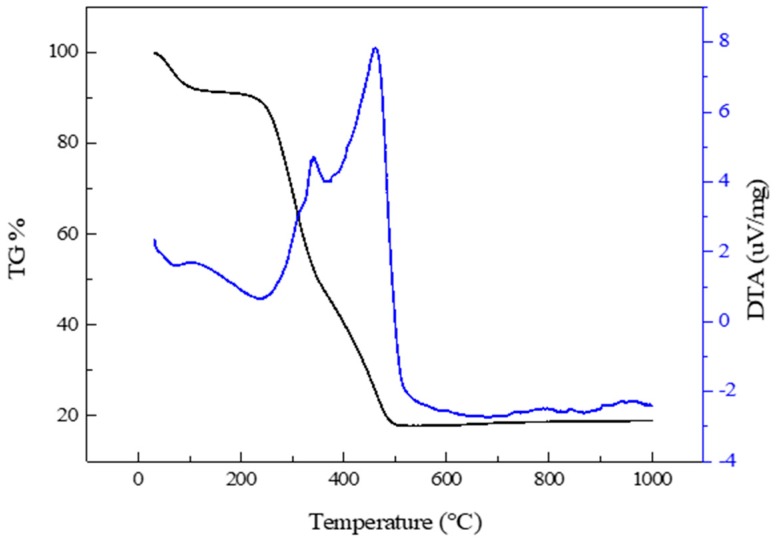
Thermogravimetric (TG), derivative thermogravimetric (DTG), and differential thermal analysis (DTA) curves of rice husk at 30–1000 °C in static air.

RH was transformed into RHA to extract the silica by eliminating organic components using thermal decomposition. The thermal properties of the rice husk were confirmed, as shown in Figure 1, by TG, DTG, and DTA. A weight loss pattern in RH was observed through the conventional thermal decomposition behaviors of the main organic components, including cellulose, hemicellulose, and lignin [14,15,16,17]. The first stage of weight loss of about 8% at 25–92 °C was due to the evaporation of moisture, and the organic constituents started to decompose from 250 °C. The rapid weight loss of 35% at 250–335 °C and the exothermic peak in DTA at 340 °C was a result of the decomposition of hemicellulose. Subsequent weight loss of about 35% at 335–520 °C with a DTG peak at 460 °C was mainly due to the decomposition of cellulose. Lignin was decomposed slowly over a broad range of temperatures from 250 to 520 °C. There was no remarkable change in the TG, DTG, and DTA curves at temperatures above ~520 °C. According to the results of the thermal analysis, a calcination temperature of 600 °C was selected to produce the RHA. This would ensure all the organic components are eliminated and only the silicate matter would remain.

The chemical composition of the RHA was examined using XRF. As indicated in Table 1, the RHA obtained through calcination of the rice husk at 600 °C was mostly silica (SiO_2_). Silica accounts for about 92% of the mass of the RHA, and high purity silica was therefore achieved following the removal of organic components in the calcination step. The main impurities were K_2_O (3.16%) and C (1.53%), while small portions of other oxides, such as CaO, P_2_O_5_, SO_3_, N, MgO, MnO, and F, were also present in the RHA. The rest of the RHA, marked as “Others” in Table 1, contained trace amounts of Al_2_O_3_, SO_3_, Cl, Cr_2_O_3_, Fe_2_O_3_, NiO, CuO, ZnO, Rb_2_O, SrO, ZrO_2_, SnO_2_, and WO_3_.

**Figure 2:**
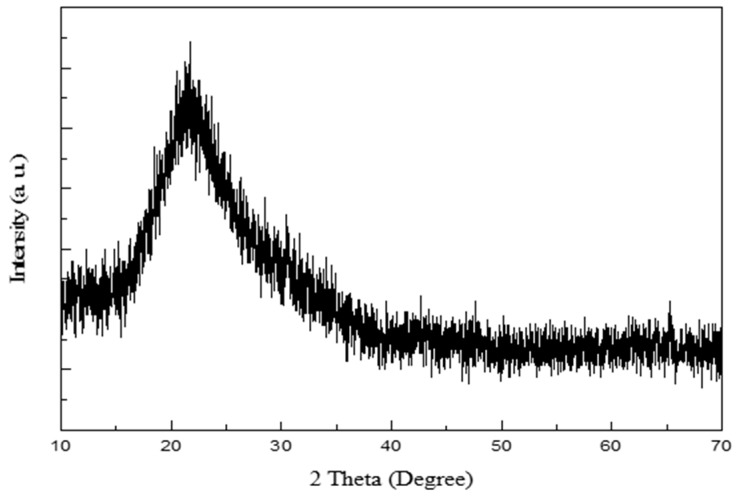
XRD pattern of the rice husk ash obtained through calcination of rice husk at 600 °C.

The XRD pattern of RHA was confirmed as a broad peak from 16° to 40° with a centered peak at 21.6°, as shown in Figure 2; this is the typical graph of amorphous silica [18]. 

The silica in the RHA was extracted in the form of sodium silicate by reaction with sodium hydroxide as follows [19,20]:

(1) $${\mathrm{SiO}}_{2}+2\;\mathrm{NaOH}\;\to {\mathrm{Na}}_{2}{\mathrm{SiO}}_{3}+H_{2}O$$

Concentrations of sodium and silicon in the prepared sodium silicate were investigated using ICP-AES, as shown in Table 2. The concentration of Si corresponded to 6.29 wt% of SiO_2_. This amount is comparable to that in an industrial water glass used for the synthesis of silica aerogel in earlier studies [21,22] and therefore represents cost-effective potential for practical applications.

To test the effect of different pH on the textural properties of silica aerogels, silica sol obtained from the sol–gel process was investigated using nitrogen physisorption. The acidity of the sol ranged between pH 1 and pH 9. The feed rate of HCl for pH adjustment was fixed at 1.5 mL/min, and other experimental conditions were kept constant to identify the effect of sol pH on the textural properties.

**Figure 3:**
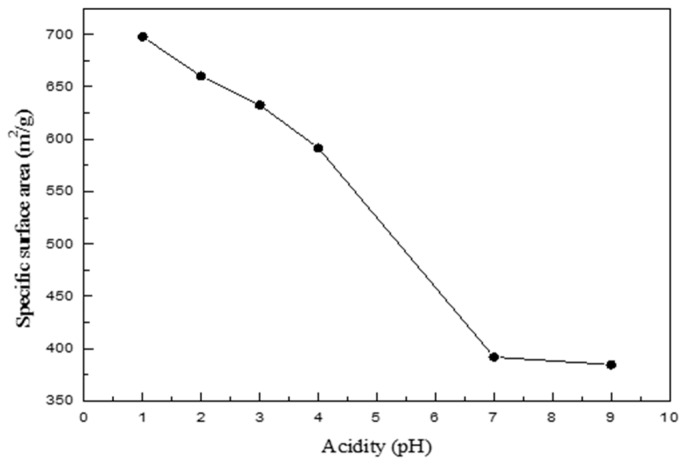
Variation in the specific surface area of the synthesized silica aerogels depending on the pH level.

The specific surface areas, average pore diameters, and pore volumes of the aerogels obtained at various pH levels are indicated in Table 3. The specific surface area, shown separately in Figure 3, increased as the pH of the sol decreased. At pH 1, the highest specific surface area of 698 m^2^/g was observed, while the pore size tended to decrease with the decrease in the sol pH, with only minor differences in the pore volumes.

The pH value, which is the major parameter in the sol–gel process, relates to the rate of hydrolysis and condensation of tetra alkoxysilanes [Si(OR)_4_] [23]. The hydrolysis reaction occurs first in the sol state under acidic condition, and the condensation reaction has the role as the rate-determining step. The reaction of terminal silicon atoms is preferred in the acidic conditions due to the electronic property, resulting in monomers or oligomers with reactive Si–OH groups at the same time. This leads to the formation of polymeric gels, which consist of clusters that condense to produce a network with small pores. Conversely, the condensation reaction is favored under alkali condition, and the hydrolysis plays a decisive role as the rate-determining step. The hydrolyzed substance is immediately consumed with the fast condensation reaction. The reaction between condensed clusters rarely takes place in the absence of inversion of configuration at one of the silicon atoms involved in the condensation reaction. Therefore, the cluster grown from the condensation of the monomer has a network structure with large pores, such as in colloidal gels [22].

As shown in Figure S1, N_2_ sorption isotherms of the aerogels exhibited a Type IV isotherm, which is typical for mesoporous materials. The silica aerogels prepared under various pH levels during the sol–gel process showed narrow pore size distributions, as shown in Figure S2.

The effect of the feed rate of hydrochloric acid, which was used for pH adjustment in the sol–gel process, on the textural properties of the aerogels was examined. The feed rate was controlled from 0.5 to 5.0 mL/min. The acidity level of silica sol was fixed at pH 1 as this pH had led to the largest specific surface area in the above experiment.

**Figure 4:**
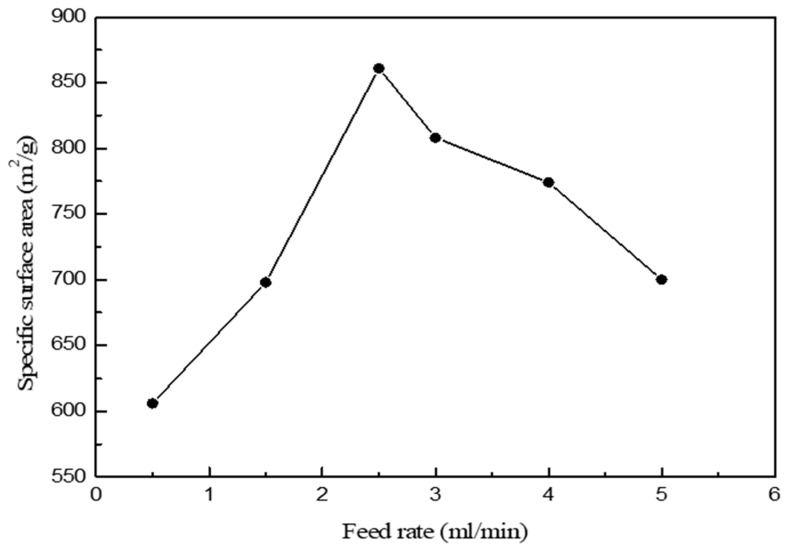
Change in the specific surface area of the silica aerogels depending on the acid feed rate.

Results showed that the specific surface area decreased at feed rates above 2.5 mL/min (Table 4). A maximum pore volume of 3.33 cm^3^/g was observed at 2.5 mL/min. The pore size showed no clear correlation with the acid feed rate. The specific surface area, as shown in Figure 4, increased with the feed rate range from 0.5 to 2.5 mL/min, with the highest specific surface area of 861 m^2^/g at 2.5 mL/min feed rate.

**Figure 5:**
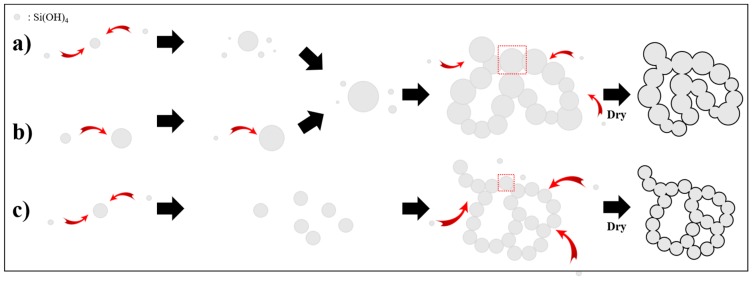
Schematic representation of particle growth and gelation at (**a**) slow, (**b**) fast, and (**c**) optimized acidic feeding rate.

Monosilicic acid surrounding the acidic droplet in a sodium silicate solution is hydrolyzed into a monomer when HCl is fed to the sodium silicate solution. The initial monomer that is aggregated restructures as large particles for stability by consuming primary smaller particles by dissolution and reprecipitation, which is termed as the Ostwald ripening mechanism [24,25]. The clusters prepared under high-acidity conditions and a slow feed rate were restructured as a colloidal consisting of a large particle as there was sufficient time to grow (Figure 5a). The pore inside of the colloidal gel was shrunk by a drying process, causing a low specific surface area. In contrast, at a fast feed rate, the initial particle consumed the smaller primary one and formed a colloidal gel with big particles and a low specific surface area, as shown in Figure 5b. However, a feed rate of 2.5 mL/min was the rate at which each cluster grew to form a particle of the same size as that resulting from the solubility equilibrium. This was due to necks developing between particles from dissolution and reprecipitation. The gel shrunk less when it dried, with enhanced stiffness and high specific surface area, as shown in Figure 5c. The morphology of each sample depending on the acidic feed rate was confirmed by FE-SEM analysis, as shown in Figure S5. The silica aerogel prepared at the slow or fast rate of acidic feeding showed shrinkage between the pore space after the drying step and had polysized particles, while the sample at optimized rate had monosized particles and large pore space.

The Type IV isotherms for the aerogels, shown in Figure S3, confirmed their mesoporous characteristics. All aerogels synthesized using various acid feed rates showed narrow pore size distributions, as shown in Figure S4.

**Figure 6:**
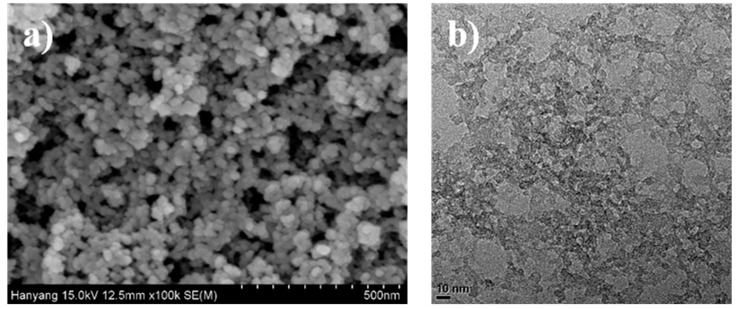
(**a**) Field emission scanning electron microscopy (FE-SEM) images of the rice-husk-based silica aerogel at a magnification of ×100,000; (**b**) transmission electron microscopy (TEM) images at a magnification of ×100,000.

The silica aerogel with optimized textural properties was obtained using silica sol with a pH 1 and an acid feed rate of 2.5 mL/min. The microstructure and morphology of the aerogel were observed using FE-SEM and TEM. As shown in Figure 6, the three-dimensional mesoporous structure of the aerogel consisted of evenly dispersed pores, and uniform particles 20 nm in diameter were identified using FE-SEM. The highly porous network of the silica aerogel was also confirmed by TEM analysis, as shown in Figure 6. The results of FE-SEM and TEM indicated that silica aerogel with well-developed porous structure was successfully formed, and a mesoporous structure was maintained even after the drying process at ambient pressure.

**Figure 7:**
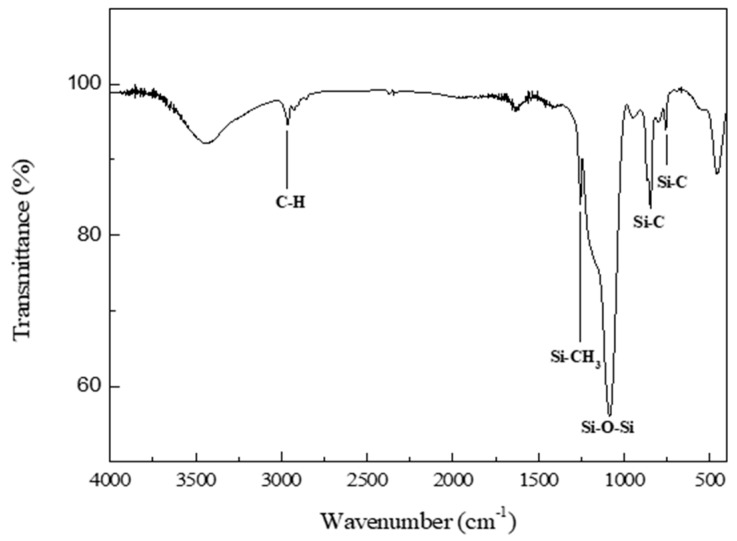
FTIR spectrum of the surface-modified silica aerogel.

FTIR was performed to verify the major functional groups and surface modification of the silica aerogel with the best textural properties (Figure 7). Peaks at 1260, 850, 760, and 2960 cm^−1^ were due to the Si–CH_3_, Si–C, Si–C, and C–H bonds, respectively. These bonds were the result of the surface modification of the silica aerogel caused by replacing the H of Si–OH on the surface with Si–CH_3_ using TMCS, thereby forming (CH_3_)_3_SiCl. As a result, the hydrophilic surface of the aerogel became hydrophobic because of the Si–CH_3_ groups. Shrinkage of the gel structure during drying and deterioration of the silica aerogel structure caused by moisture adsorption can be prevented by the hydrophobicity of the aerogel. A very intense peak at 1080 cm^−1^ was attributed to the siloxane linkages (Si–O–Si), and a broad peak at 3450 cm^−1^ was due to the remaining silanol groups. In the surface modification process using TMCS as a silylating agent, the following chemical reactions are expected [22,26,27,28]:

(2) $${\left({\mathrm{CH}}_{3}\right)}_{3}-\mathrm{Si}-\mathrm{Cl}\;\left[\mathrm{TMCS}\right]+\equiv \mathrm{Si}-\mathrm{OH}\to \equiv \mathrm{Si}-O-\mathrm{Si}-{\left({\mathrm{CH}}_{3}\right)}_{3}+\mathrm{HCl}$$

(3) $$2{\left({\mathrm{CH}}_{3}\right)}_{3}-\mathrm{Si}-\mathrm{Cl}+H_{2}O\to {\left({\mathrm{CH}}_{3}\right)}_{3}-\mathrm{Si}-O-\mathrm{Si}-{\left({\mathrm{CH}}_{3}\right)}_{3}\left[\mathrm{HMDSO}\right]+2\mathrm{HCl}$$

(4) $${\left({\mathrm{CH}}_{3}\right)}_{3}-\mathrm{Si}-\mathrm{Cl}+C_{2}H_{5}\mathrm{OH}\to {\left({\mathrm{CH}}_{3}\right)}_{3}-\mathrm{Si}-O-{\mathrm{CH}}_{2}{\mathrm{CH}}_{3}\left[\mathrm{ETMS}\right]+\mathrm{HCl}$$

(5) $$2{\left({\mathrm{CH}}_{3}\right)}_{3}-\mathrm{Si}-O-{\mathrm{CH}}_{2}{\mathrm{CH}}_{3}+H_{2}O\to {\left({\mathrm{CH}}_{3}\right)}_{3}-\mathrm{Si}-O-\mathrm{Si}-{\left({\mathrm{CH}}_{3}\right)}_{3}+2C_{2}H_{5}\mathrm{OH}$$

TMCS reacts with the surface silanol groups (Equation (2)), water in the pores (Equation (3)), and intermediate solvent, ethanol (Equation (4)). The reactions with water and ethanol produce hexamethyldisiloxane (HMDSO) and ethoxytrimethylsilane (ETMS), respectively. ETMS reacts with water, as indicated in Equation (5).

**Figure 8:**
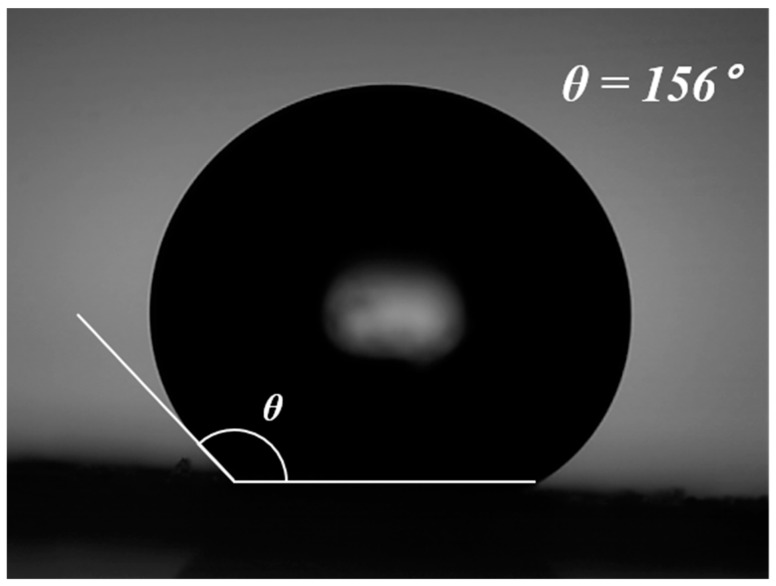
Water contact angle (θ) on the hydrophobic surface of the silica aerogel.

The hydrophobicity of the silica aerogel was quantified by the contact angle (θ) measurement. As shown in Figure 8, a water droplet on the hydrophobic surface of the silica aerogel exhibited a contact angle of 156°. Nonpolar alkyl groups on the aerogel surface make the surface hydrophobic and rough [28,29]. The droplet can hardly enter inside the highly hydrophobic surface and form a compact shape. A contact angle higher than 150° means almost no contact between the surface and the liquid. Thus, the surface is nonwettable. The hydrophobic surface reduces the surface tension caused by evaporation of the pore liquid. The decrease in the surface tension thus results in a silica aerogel with high porosity, low density, and high transparency.

**Figure 9:**
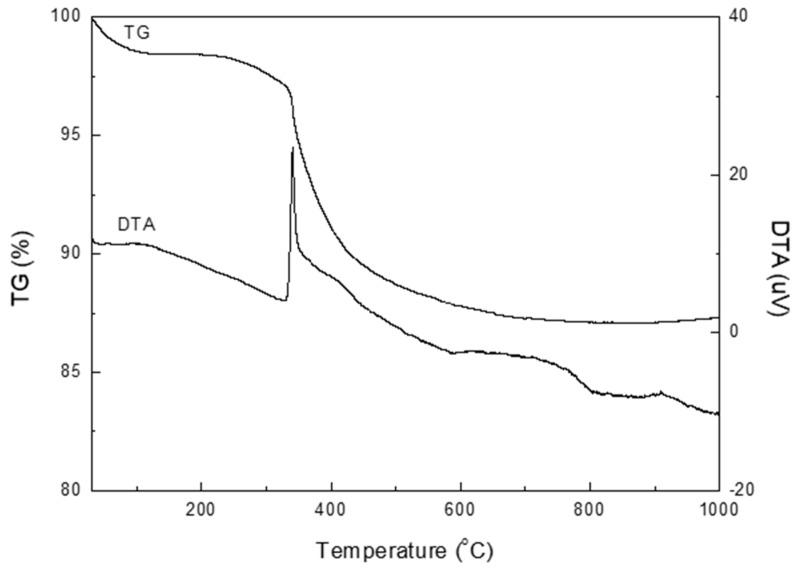
TG and DTA curves of the silica aerogel at 30–1000 °C in air.

The thermal stability and retention of hydrophobicity of the silica aerogel were analyzed by TG and DTA. The TG and DTA curves of the aerogel at 30–1000 °C in air are shown in Figure 9. A slight weight loss from 30–100 °C in the TG curve was due to the evaporation of moisture. The weight loss of 9% at 340–700 °C was attributed to oxidation of the surface methyl groups, which made the aerogel surface hydrophobic [7,26]. A sharp exothermic peak at 340 °C in the DTA curve indicated oxidation. The silica aerogel lost its hydrophobicity and became hydrophilic at temperatures above 340 °C. To confirm the loss of hydrophobicity, the aerogel was calcined at 450 °C. The surface of the calcined aerogel was completely wet once a drop of water was placed on the aerogel surface. The TG curve showed no significant change at temperatures over ~700 °C.

## 4. Conclusions

Rice husk, a biomass resource left after threshing rice, is considered as a sustainable and economical raw material for large-scale production of silica aerogel due to its high silica content. In this research, control of the specific surface area of silica aerogel from rice husk under a relatively simple process was conducted with an eco-friendly, re-usable, low-cost and safe approach compared to the conventional method. Silica aerogel was prepared by amorphous silica extracted in the form of sodium silicate from rice husk ash, and the silicate was then neutralized to form silica gel via the sol–gel process. The silica gel became hydrophobic aerogel through solvent exchange and surface modification at ambient pressure. The porosity of aerogel was controlled by adjusting the pH and the acid feed rate in the sol–gel process; these parameters contributed to regulating the silica particle growth. The sample by titration until approaching pH 1 at 2.5 mL/min showed the best textural properties of 861.2280 m^2^/g. The aerogel was highly hydrophobic with a water contact angle of 156°, and the hydrophobicity was retained up to ~340 °C. These results imply that aerogels from rice husk with regulated porosity has the potential to be applied for mass production in an economic manner.

## Figures and Tables

**Table 1:** Oxide composition of the rice husk ash (RHA).

Oxide	Mass %
SiO_2_	92.07
K_2_O	3.16
C	1.53
CaO	0.76
P_2_O_5_	0.66
SO_3_	0.56
N	0.27
MgO	0.24
MnO	0.21
F	0.20
Others	0.35

**Table 2:** Concentrations of Na and Si in the sodium silicate from the rice husk ash.

Sample	Na (ppm)	Si (ppm)
Sodium silicate	22,808.75	29,586.03

**Table 3:** Textural properties of the silica aerogels depending on the sol pH.

pH	Specific Surface Area (m^2^/g)	Mean Pore Diameter (nm)	Pore Volume (cm^3^/g)
1	697.7559	15.9180	2.7767
2	660.1251	17.6105	2.9062
3	632.4830	15.9664	2.5246
4	591.4873	18.6045	2.7510
7	391.9549	30.3362	2.9726
9	384.6501	28.5409	2.7445

**Table 4:** Textural properties of the silica aerogels according to acid feed rate.

Feed Rate (mL/min)	Specific Surface Area (m^2^/g)	Mean Pore Diameter (nm)	Pore Volume (cm^3^/g)
0.5	606.2524	15.8509	2.4024
1.5	697.7559	15.9180	2.7767
2.5	861.2280	15.6587	3.3714
3.0	808.4978	12.5028	2.5271
4.0	774.2045	13.1030	2.5361
5.0	700.5603	15.8025	2.7676

## Supplementary Materials

The following are available online at http://www.mdpi.com/2079-4991/9/2/300/s1, Figure S1: Nitrogen adsorption isotherms of the silica aerogels obtained at various values of pH, Figure S2: Pore size distributions of the silica aerogels prepared at various pH, Figure S3: Nitrogen adsorption isotherms of the silica aerogels synthesized using various acid feed rates, Figure S4: Pore size distributions of the silica aerogels obtained using various acid feed rates, Figure S5: FE-SEM images of the rice husk-based silica aerogel at the acidic feed rate of 0.5~5.0 mL/min.
