# Supplementary material for: Effect of Acidity Levels and Feed Rate on the Porosity of Aerogel Extracted from Rice Husk under Ambient Pressure
Source: Nanomaterials (Basel). 2019 Feb 20;9(2):300. doi: 10.3390/nano9020300 (PMC6410322; doi:10.3390/nano9020300)
Supplement: Supplementary file 1 [file nanomaterials-09-00300-s001.pdf]

Supporting Information

Effect of Acidity Levels and Feed Rate on the Porosity of Aerogel Extracted from Rice Husk under Ambient Pressure

Garram Ban [1,†], Sinae Song [2,†], Hong Woon Lee [1,3] and Hee Taik Kim [1,2,*]

[1] Department of Fusion Chemical Engineering, Hanyang University, 55 Hanyangdaehakro, Sangnok-gu, Ansan, Gyeonggi-do 15588, Korea; kdtry0@naver.com (G.B.); kkongbu@daega.co.kr (H.W.L.)

[2] Department of Advanced Materials Science and Engineering, Hanyang University, 55 Hanyangdaehakro, Sangnok-gu, Ansan, Gyeonggi-do 15588, Korea; mokakid@hanyang.ac.kr

[3] Daega Powder Systems Co., LTD., Head office & Seoul factory, 22-31, Buil-ro 1na-gil, Guro-gu, Seoul-si 08262, Korea

* Correspondence: khtaik@hanyang.ac.kr; Tel.: +82-31-400-5274

† Those authors contributed equally to this work.

Figure of contents

Figure S1. Nitrogen adsorption isotherms of the silica aerogels obtained at various values of pH.

Figure S2. Pore size distributions of the silica aerogels prepared at various pH.

Figure S3. Nitrogen adsorption isotherms of the silica aerogels synthesized using various acid feed rates.

Figure S4. Pore size distributions of the silica aerogels obtained using various acid feed rates.

Figure S5. FE-SEM images of the rice husk-based silica aerogel at the acidic feed rate of 0.5 ~ 5.0 mL/min.

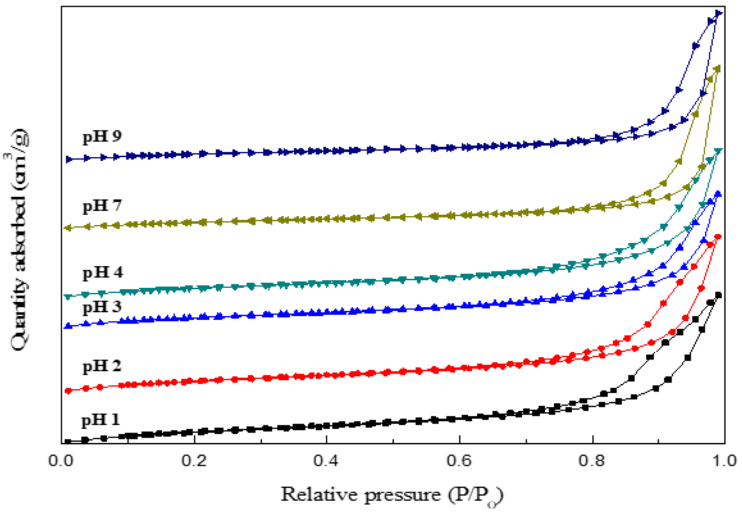

Figure S1. Nitrogen adsorption isotherms of the silica aerogels obtained at various values of pH.

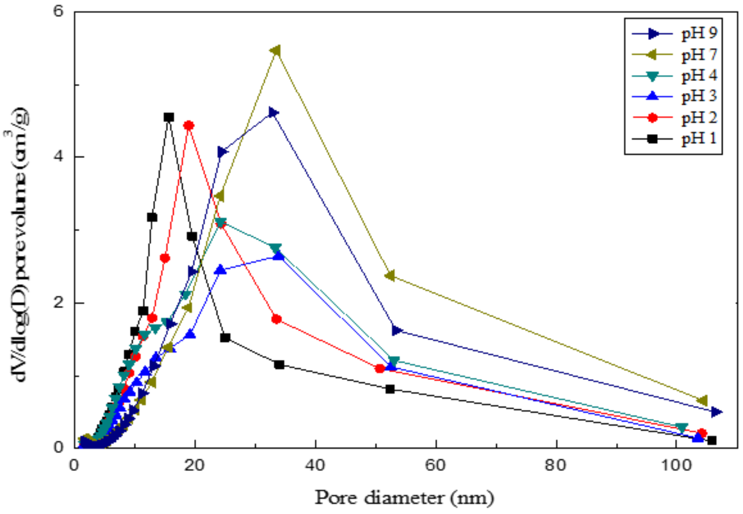

Figure S2. Pore size distributions of the silica aerogels prepared at various pH.

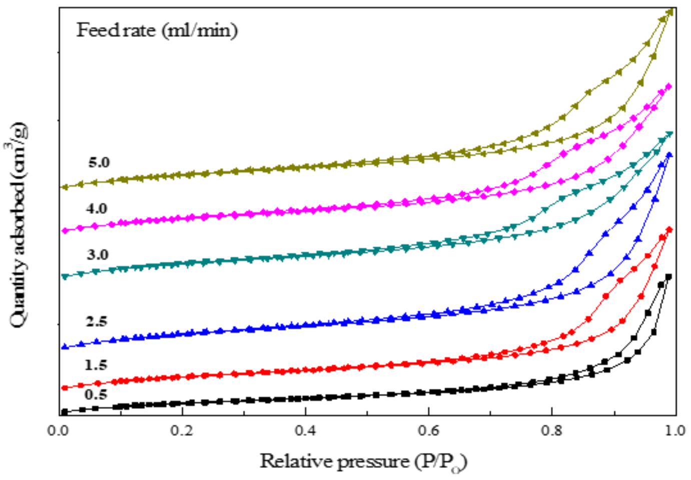

Figure S3 Nitrogen adsorption isotherms of the silica aerogels synthesized using various acid feed rates.

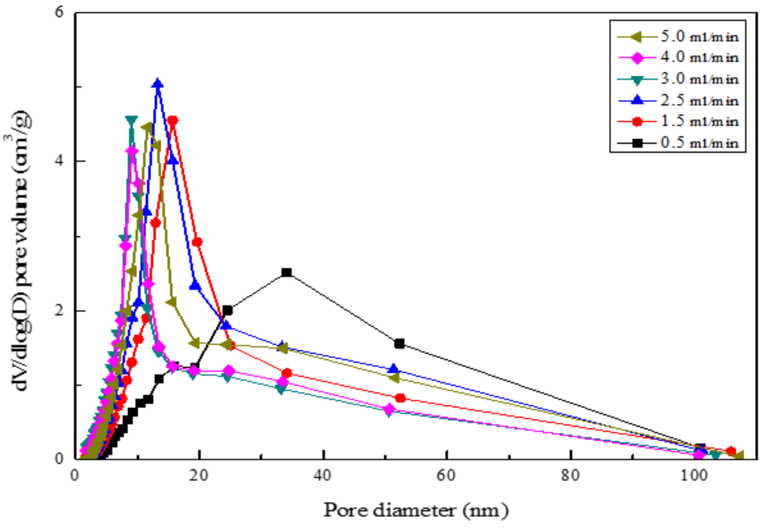

Figure S4. Pore size distributions of the silica aerogels obtained using various acid feed rates.

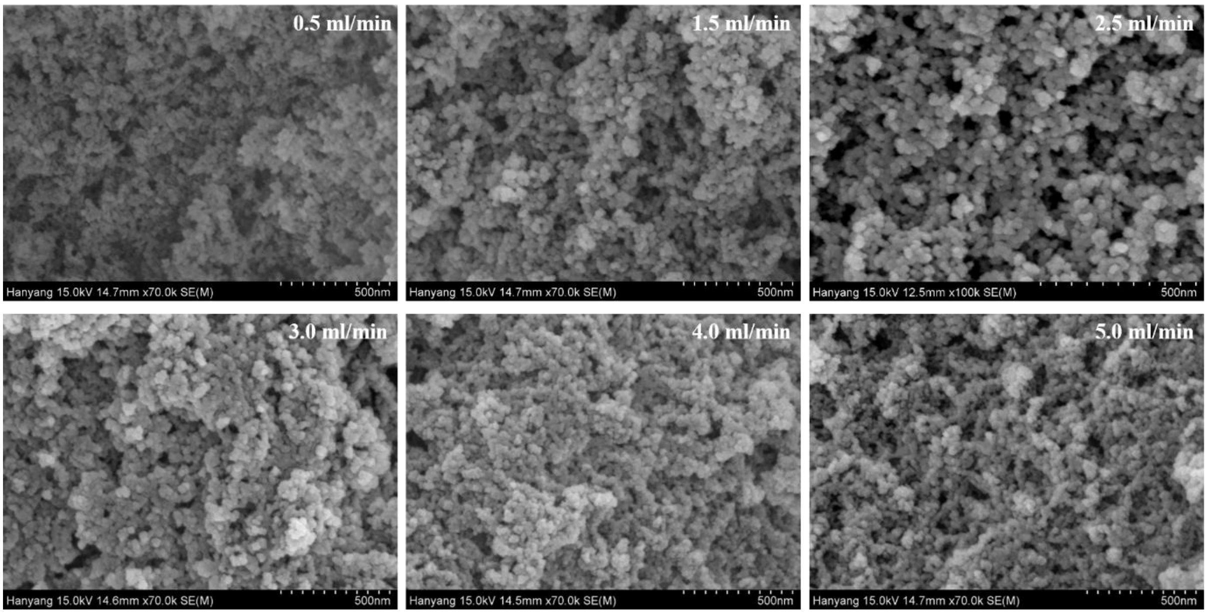

Figure S5. FE-SEM images of the rice husk-based silica aerogel at the acidic feed rate of 0.5 ~ 5.0 mL/min.
